# Collectin-11, a complement pattern recognition molecule, mediates pulmonary SARS-CoV-2 neutralization and protection

**DOI:** 10.1371/journal.ppat.1014216

**Published:** 2026-05-13

**Authors:** Adrian Sutta, Anna Offersgaard, Carlos Rene Duarte Hernandez, Tereza Alica Plchová, Yuyong Zhou, Laura Pérez-Alós, Anne Rosbjerg, Rafael Bayarri-Olmos, Jens Bukh, Beatriz González-García, Judith Margarete Gottwein, Peter Garred

**Affiliations:** 1 Laboratory of Molecular Medicine, Department of Clinical Immunology, Copenhagen University Hospital, Copenhagen, Denmark; 2 Copenhagen Hepatitis C Program (CO-HEP), Department of Infectious Diseases, Copenhagen University Hospital–Hvidovre, Hvidovre, Denmark; 3 CO-HEP, Department of Immunology and Microbiology, Faculty of Health and Medical Sciences, University of Copenhagen, Copenhagen, Denmark; 4 Department of Clinical Medicine, Faculty of Health and Medical Sciences, University of Copenhagen, Copenhagen, Denmark; Duke-National University of Singapore, SINGAPORE

## Abstract

**Background:**

Collectin-11 (CL-11) is a complement-activating pattern recognition molecule with structural and functional similarities to mannose-binding lectin (MBL), produced in different tissues, including lung epithelium. Given its tissue localization and role in innate immunity, we investigated its potential to recognize and neutralize severe acute respiratory syndrome coronavirus 2 (SARS-CoV-2) and to activate complement.

**Methods:**

We produced recombinant CL-11, MBL, as well as wild-type, variant, and glycan-mutated SARS-CoV-2 Spike (S) proteins. We evaluated CL-11 binding to the S protein, complement activation, and inhibition of S protein-receptor binding using ELISA, as well as neutralization of SARS-CoV-2 in cell-based neutralization assays.

**Results:**

CL-11 bound different SARS-CoV-2 S protein variants with similar binding preferences as MBL, targeting multiple glycan sites. Upon S protein binding, CL-11 mediated activation of both the lectin and alternative complement pathways, and inhibited S protein-receptor binding. Notably, CL-11 neutralized SARS-CoV-2, inhibiting infection of permissive cells.

**Conclusion:**

CL-11 binds different SARS-CoV-2 variants and neutralizes SARS-CoV-2 in an antibody-independent manner, suggesting a crucial role in early-stage infection control.

## Author summary

Collectin-11 (CL-11) is a key component of the lectin complement pathway with previously unrecognized antiviral properties. Its local production in lung epithelium suggests a crucial role in respiratory defense against viruses. Our study describes how CL-11 binds to SARS-CoV-2, activates complement, and neutralizes the virus in an antibody-independent manner. These findings uncover a new layer of innate immune protection against SARS-CoV-2 and provide insight into lectin complement pathway-mediated antiviral defense. Identifying CL-11 as an antiviral effector opens new avenues for developing innovative antiviral therapies in protection against respiratory infections.

## Introduction

The innate immune system is the first sentinel against pathogens, responsible for controlling and eliminating them in the early stages of infection. The complement system is the main humoral effector mechanism of the innate immunity involved in pathogen removal, immunomodulation, and homeostasis [1]. Once induced, it can recruit other immune cells, opsonize pathogens, and cause lysis in susceptible cells through its terminal membrane attack complex (MAC) [2]. The complement system can be activated via three pathways – classical, lectin, and alternative [3].

Complement activation via the lectin pathway is driven by pattern recognition molecules (PRMs), such as members of the collectin protein family, including mannose-binding lectin (MBL) and collectin-11 (CL-11, collectin kidney 1, CL-K1) [4]. MBL is a central molecule of the lectin pathway, with well-documented antiviral capabilities against respiratory viruses, [5] including Severe Acute Respiratory Syndrome Coronavirus (SARS-CoV) [6,7] and SARS-CoV-2 [8–11]. MBL attaches to high mannose and N-acetylglucosamine oligosaccharides on the surfaces of enveloped respiratory viruses, leading to opsonization, agglutination, inhibition of viral fusion, and complement activation [11].

SARS-CoV-2 is a highly contagious respiratory virus that infects both humans and animals causing Coronavirus disease 2019 (COVID-19) [2]. While most individuals experience mild to moderate symptoms, some develop severe respiratory complications, leading to critical illness and requiring intensive medical intervention, including mechanical ventilation and prolonged hospitalization [2]. SARS-CoV-2 is a single-stranded enveloped RNA virus, notable for its high mutation rate and genetic adaptability [12]. The envelope of the virus comprises three structural proteins: The envelope (E), Membrane (M), and Spike (S) proteins [13]. The S protein in particular plays a key role in SARS-CoV-2 infection, as it mediates the entry of the virus into the host cells. It is a trimeric glycoprotein with two subunits, S1 and S2. The S1 subunit is responsible for binding to the host cell through two domains: the receptor binding domain (RBD) and the N-terminal domain (NTD) [14]. NTD supports the optimal conformation of RBD for binding [14], while RBD binds directly to the main entry receptor, the host cells’ angiotensin- converting enzyme-2 (ACE-2) [15]. The surface of the S protein is heavily glycosylated with abundant N-glycans and O-glycans [16]. Glycosylation, at these highly conserved glycan sites plays a role in the proper folding and functionality of the S protein and immune evasion [17]. However, the presence of the glycans makes the S protein a potential target for PRMs of the innate immune system [11].

While MBL has been extensively studied, the role of CL-11 in viral infections is less well understood. CL-11 shares many structural and functional similarities with MBL [17]. Structurally, both proteins have a globular carbohydrate recognition domain (CRD) at the C-terminal part of the protein involved in ligand recognition, a coiled-coil alpha-helical neck region, a collagenous domain, and a small N-terminal region [17]. Through the neck region, three monomers can be assembled into a trimer - the smallest functional subunit. The N-terminal region contains a single cysteine residue that allows the trimers to organize into higher-order oligomers of trimers such as tetramers of trimers [18]. The formation of higher-order oligomeric structures is crucial for the binding avidity and effector functions of the collectins [19]. The collagen-like region influences the effector functions of the collectins by facilitating their interaction with a set of associated serine proteases termed MASP-1, -2, and -3 [20]. MASP-1 activates MASP-2, and the latter cleaves complement cascade proteins C4 and C2 leading to the formation of the C3 convertase through the lectiathway [21]. MASP-3 activates Factor D, and thus regulates the activation of the alternative pathway [22]. However, one of the key differences between CL-11 and MBL is their tissue distribution. While MBL is produced exclusively in the liver and released into the circulation, CL-11 is produced locally by multiple cell types, including lung tissue cells [23,24]. This suggests that CL-11 may play a critical role in the local defense against respiratory viruses, as it can be released directly onto epithelial surfaces where these viruses typically enter the body. A genome-wide study by Ramlall et al. in 2020 reported an association between COVID-19 severity and CL-11 levels, suggesting that variations in CL-11 levels and activity may influence disease outcomes [25].

Based on these observations, we hypothesized that CL-11 can bind to the S protein of SARS-CoV-2, activate the complement system, and neutralize the virus in an antibody-independent manner, highlighting the relevance of CL-11 in the defense against respiratory viruses.

## Materials and methods

### Production and purification of recombinant proteins

Recombinant (r) CL-11 (NM_024027.5) was produced by transient transfection using the ExpiCHO Expression System Kit (Thermo Fisher Scientific, Waltham, Massachusetts, USA). Briefly, ExpiCHO cells grew shaking in suspension in a humidified incubator at 37°C with 8% CO_2_ and were transfected following the Max Titer Protocol, as described in the manufacturer’s instructions. On day 8 after transfection, the cell culture supernatant was harvested and clarified by centrifugation for 5 min at 1000 x g. Protein content of the supernatant was verified and quantified using an in-house established sandwich Enzyme-Linked Immunosorbent Assay (ELISA) [26]. The presence of higher-order complexes of CL-11 was confirmed by Western blot (WB) **(Fig A.A** in **S1 Text**). The SARS-CoV-2 RBD, S1 N-terminal domain (NTD), ACE-2, and rMBL proteins were produced and purified as described previously [27–29]. The purity and molecular weight of the proteins were assessed by Coomassie Blue staining **(Fig A.B** in **S1 Text**).The full-length Wild Type (WT) SARS-CoV-2 S protein (NC_045512.2:21563–25384; YP_009724390.1) and the S proteins of the four Omicron variants BA.5, BA.2.75, XBB.1.5, and BQ.1.1 (mutations from https://covariants.org/ and https://outbreak.info/) were produced by transient expression in ExpiCHO cells using the method described for CL-11. The sequence of the extracellular domain of the S proteins was codon optimized for *Cricetulus griseus* with the following modifications: the change of the furin cleavage site (682RRAR685–682GSAS685) to avoid the cleavage of S1 and S2 subunits, the addition of a hexaPro motif (K986P, V987P, F817P, A892P, A899P, A942P) for the stabilization of the proteins, a foldon domain for the proper folding and stabilization as trimers, a His-tag (8xHis) for their purification, and an Avi-tag for specific biotinylation of the proteins. The recombinant S proteins were His-tag purified from the supernatant using Ni-NTA beads (HisPur Ni-NTA Resin, Thermo Fisher Scientific) with further separation by affinity chromatography. Recombinant proteins were eluted by competition with 250 mM imidazole. The eluted proteins were dialyzed in phosphate-buffered saline (PBS) buffer to remove the imidazole (56750, Sigma-Aldrich, St. Louis, Missouri, USA) and kept at -20°C until use in experiments. The S proteins containing glycan mutations in their sequence were generated by site-directed mutagenesis by GeneArt (Thermo Fisher Scientific). 11 N-glycan sites within the S protein were identified and introduced into the S protein sequences to generate single, double, or triple mutants as described elsewhere [29]. rMASP-2 and rMASP-3 were produced as described previously [30,31]. rPro-Factor D (NM_001928.4:27–788) was generated through transient transfection of Expi293 cells, using the Expi293 Expression System Kit (Thermo Fisher Scientific). The cells were cultured in suspension on a shaker in a humidified incubator set at 37°C with 8% CO₂. Transfection was performed according to the manufacturer’s Protocol and supernatant was collected 7 days post-transfection.

## rCL-11 binding experiments

### Binding to carbohydrate ligands and WT S protein

In a direct ELISA setup, MaxiSorp 96-well microtiter plates (439454, Thermo Fisher Scientific) were coated overnight (ON) at 4°C with carbohydrate ligands mannan (M7504, Sigma-Aldrich) or zymosan A (Z-4250, Sigma-Aldrich) and WT S protein at a constant concentration of 2 µg/ml. Then, purified rMBL or rCL-11 supernatant was added in serial 2-fold dilutions starting with 1 µg/ml and incubated for 1.5 h in tris-buffered saline (TBS-Tween-20 (Tw); 10 mM Tris, 150 mM NaCl + 0.05% Tw (655204, Millipore, Burlington, Massachusetts, USA) + 2.5 mM CaCl₂, pH 7.4). rCL-11 was detected with monoclonal antibody (mAb) hybridoma (HYB) 15 biotinylated (*in-house*^*26*^, 2 µg/ml in PBS-Tw) and MBL with HYB 131-1 biotinylated (*in-house*^*32*^, 2 µg/ml in PBS-Tw) for 1.5 h in dilution buffer. For the final detection, streptavidin-Horseradish Peroxidase (HRP) conjugate (RPN1231V Cytiva, Marlborough, Massachusetts, USA) was incubated for 1 h (1:2000 in PBS-Tw). TMB ONE (KemEnTec Diagnostics, Taastrup, Denmark), was used as substrate for the colorimetric enzymatic reaction, which was stopped with 0.3 M H_2_SO_4_, and optical density (OD) was recorded at 450 nm with 630 nm wavelength correction using a Biotek plate reader (Agilent, Santa Clara, California, USA). Plates were washed three times in TBS-Tw between each incubation step and four times in the last step. Incubations were performed at room temperature (RT) on an orbital shaker at 550 rotations per minute.

### Binding to NTD, RBD, and WT S protein

MaxiSorp 96-well microtiter plates (439454, Thermo Fisher Scientific) were coated ON at 4°C with WT full-length S protein, S1 NTD, and RBD. The WT full-length S protein was added at a concentration corresponding to 2 µg/ml or 11.1 pM, and NTD and RBD were normalized to the same molar concentration (11.1pM). Next, 0.5 µg/ml of rCL-11 ± EDTA (E-5134, Sigma-Aldrich) in TBS-Tw was added and incubated for 1.5 h at RT. Bound rCL-11 was detected with HYB 15 biotinylated and streptavidin-HRP, and plates were washed and developed as described above.

### Binding to S protein variants

The full-length S proteins from SARS-CoV-2 WT and from the four Omicron variants BA.5, BA.2.75, XBB.1.5, and BQ.1.1, were coated onto the MaxiSorp 96-well microtiter plate (439454, Thermo Fisher Scientific) in serial 3-fold dilutions starting at 1 µg/ml and incubated ON at 4°C. Subsequently, rCL-11 was added to the S proteins at a constant concentration of 0.5 µg/ml in dilution buffer for 1.5 h at RT. Bound rCL-11 was detected with HYB 15 biotinylated and streptavidin-HRP, and plates were washed and developed as described above.

### Binding to S protein glycan site mutants

MaxiSorp 96-well microtiter plates (439454, Thermo Fisher Scientific) were coated with HYB 53 anti-RBD mAb (*in-house*, [28], 2 µg/ml in PBS) ON at 4°C. Following the coating with antibody, WT S protein supernatants with N-glycan mutations in their sequence were added at 1 µg/ml and incubated for 2 h at RT. Subsequently, rCL-11 supernatant was added at 0.5 µg/ml in TBS-Tw for 1.5 h at RT. Bound rCL-11 was detected with HYB 15 biotinylated and streptavidin-HRP, and plates were washed and developed as described above.

## rCL-11-mediated complement deposition

### Deposition of recombinant C4 on WT S protein

MaxiSorp 96-well microtiter plates (439454, Thermo Fisher Scientific) were coated with WT S protein and mannan in PBS at 5 µg/ml ON at 4°C. Then, 10 µg/ml of rCL-11 supernatant, ± EDTA, was incubated for 1.5 h at RT. Subsequently, 1 µg/ml of purified rMASP-2, ± EDTA, was added and left to incubate for 2 h in dilution buffer while shaking at 37°C, followed by addition of purified C4 (CompTech, Tyler, Texas, USA) at 0.2 µg/ml for 30 min while shaking at 37°C. C4 was detected using polyclonal anti-human C4c biotinylated antibody (Q036905-2, Agilent) at 1 µg/ml in PBS-Tw incubated while shaking for 1 h at RT. The plates were incubated with streptavidin-HRP, washed, and developed as described above.

### Deposition of C4, C5 and terminal complement complex (TCC) from serum on WT S protein

MaxiSorp 96-well microtiter plates (439454, Thermo Fisher Scientific) were coated with WT S protein at 2 µg/ml in PBS ON at 4°C. After coating, rCL‑11 supernatant was added as a 2‑fold dilution series in Barbital‑Tw buffer (4 mM sodium barbital, 145 mM NaCl, 2.6 mM CaCl₂, 2.1 mM MgCl₂, 0.05% Tw, pH 7.4) starting at 8 µg/ml and incubated for 1.5 h at RT. MBL‑deficient serum diluted to 2% or 10% in Barbital‑Tw was then added and incubated with shaking at 37°C for 1 h. Deposition of complement components was detected using specific monoclonal antibodies: anti-C3bc (in-house produced, clone bH6) [32,33], anti-C4b (biotinylated Hyb 162–02, Bioporto Diagnostics), anti-TCC (in-house produced, clone aE11) [34,35], each at 2 µg/ml in Barbital-Tw, followed by 1.5 h incubation at RT with shaking. Primary antibodies were detected using either streptavidin‑HRP or a polyclonal rabbit anti‑mouse HRP‑conjugated secondary antibody (P0260 Dako, Agilent, Santa Clara, CA, US), diluted 1:2000 in Barbital-Tw and incubated for 1 h at RT with shaking. The plates were washed (Barbital-Tw) and developed as described above.

### CL-11-mediated activation of Factor D

MaxiSorp 96-well microtiter plates (439454, Thermo Fisher Scientific) were coated with 1 µg/ml solution of WT S protein and incubated at 4°C ON. The next day, the plates were blocked with PBS-Tw for 30 minutes and incubated with rCL-11 supernatant at 1 µg/ml, for 1 h at RT in Barbital-Tw. Subsequently, 1 µg/ml of either active rMASP-3, zymogen rMASP-3 in Barbital-Tw, or just buffer was added, followed by incubation for 1 h at RT. A total of 100 µl of rPro-Factor D supernatant was added to each well and incubated at 37°C while shaking for 0, 2, 6, or 24 h. Following incubation, 9 µl of supernatant from each well was collected and analyzed by SDS-PAGE using a 4–12% Bis-Tris gel (NP0321, Thermo Fisher Scientific) with MES buffer, with 0.25 µg of Factor D (A136, CompTech,) added in the first lane as a loading control. The proteins were then transferred onto a nitrocellulose membrane (LC2001, Thermo Fisher Scientific) for WB analysis. Detection of Factor D was performed using 0.2 µg/ml of a mouse mAb anti-Factor D (clone Act 17, *in-house*^35^), which specifically recognizes mature Factor D but not pro-Factor D. Additionally, 0.1 µg/ml in*-house* antibody recognizing both pro-Factor D and mature Factor D was used (clone F1-11, *in-house*^35^). A rabbit anti-mouse HRP-conjugated (P0260, Agilent) was used as a secondary antibody, applied at a concentration of 0.1 µg/ml in PBS. Plates were washed and developed as described above. The bands were quantified by LI-COR Odyssey Fc (LI-COR Inc., Lincoln, Nebraska USA), and the activation was calculated as the percentage of the Active Factor D band signal intensity compared to the Total Factor D band signal intensity at the 0, 2, 6, and 24 h timepoints.

## ELISA-based S protein-receptor binding assay

MaxiSorp 96-well microtiter plates (439454, Thermo Fisher Scientific) were coated with 1 µg/ml of ACE-2 ON at 4°C. rCL-11 or rMBL in serial 2-fold dilutions starting at 20 µg/ml in dilution buffer were co-incubated in a dilution plate (249944, Thermo Fisher Scientific) with 1 µg/ml of WT S protein and incubated for 1 h while shaking at RT. After the incubation in the fluid phase, supernatant from the dilution plates were added to the ACE-2-coated microtiter plate and incubated for 1 h at RT. Bound S protein was detected with HYB 46 biotinylated mAb (*in-house*^*28*^, 2 µg/ml TBS-Tw). The plate was incubated with streptavidin-HRP, washed, and developed as described above.

## Cell culture-based virus neutralization assay

### Cell lines

The African green monkey kidney cell line VeroE6 was maintained as described previously [36,37] The cells were cultivated in Dulbecco’s Modified Eagle Medium (DMEM) (Thermo Fisher Scientific) supplemented with 10% fetal bovine serum (FBS) (Sigma-Aldrich), 100 U/ml penicillin, and 100 µg/ml streptomycin (Sigma-Aldrich) at 37°C and 5% CO_2_ and sub-cultured every 2–4 days. The cells were detached with Trypsin (Sigma-Aldrich).

The human lung adenocarcinoma A549-hACE-2 cell line (InvivoGen, San Diego, California, USA) (A549 cells expressing human ACE-2) was maintained as described previously [36]. The cells were cultivated in DMEM: Nutrient Mixture F-12 (Thermo Fisher Scientific) supplemented with 10% FBS, 100 U/ml penicillin, 100 µg/ml streptomycin, and 0.5 µg/mL puromycin (InvivoGen) at 37°C and 5% CO₂ and sub-cultured every 2–3 days. The cells were detached with Trypsin.

The human lung adenocarcinoma Calu-3 cell line was maintained as described previously [37]. The cells were cultivated in Eagle’s Minimum Essential Medium supplemented with 10% FBS, 100 U/ml penicillin, and 100 µg/ml streptomycin at 37°C and 5% CO_2_ and sub-cultured every week [38]. The cells were detached with Trypsin.

The human hepatoma Huh7.5 cell line was maintained as described previously [39]. The cells were cultivated as described for the VeroE6 cells.

### SARS-CoV-2 virus

Original SARS-CoV-2 virus with the D614G mutation in the S protein (SARS-CoV-2/human/DNK/DK-AHH1/2020; GenBank: MZ049597) was used throughout the study. The virus was isolated from a patient as described previously [37], and a passage 2 stock was used in cell culture-based experiments [38]. The infectious titer of the virus stock was determined as described in the section ”virus infectious titers” and used to calculate MOI in virus neutralization assays.

### Virus infectious titers

The infectious titer of the virus was determined as described previously [37,40]. Briefly, 96-well plates (Thermo Fisher Scientific) were seeded with 10,000 VeroE6 cells/well and incubated at 37°C and 5% CO_2_. The next day, serial dilutions of viral samples were prepared in supplemented DMEM, and 100 µl of each dilution were added to quadruplicate wells. After 48 ± 2 h incubation, the plates were fixed and virus was inactivated in cold methanol for 20 min followed by immunostaining using primary anti-S antibody (AB_2827983, Sino Biological, Beijing, China) diluted 1:5000 in BSK (PBS containing 1% bovine serum albumin and 0.2% skimmed milk) and incubated for 2 h at RT. The primary antibody was detected using F(ab’)2-Goat anti-Human IgG Fc Cross-Absorbed-HRP conjugated secondary antibody (AB_2535945, Thermo Fisher Scientific) diluted 1:2000 in BSK and incubated 1 h at RT. SARS-CoV-2 positive cells were visualized using the Bright-DAB solution kit (Immunologic, Duiven, Netherlands), and each of the plates was imaged using the Immunospot series 5 UV analyzer (CTL Europe GmbH, Rutesheim, Germany) [41]. Finally, the 50% tissue culture infectious dose (TCID_50_) was determined according to the Reed-Muench method [40].

### Direct virus neutralization assay

In the conventional experimental setup, 10,000 cells/well were seeded in 96-well plates the day prior to the experiment for VeroE6, A549-hACE-2, and Huh7.5 cells. For Calu-3 cells, 25,000 cells/well were seeded 4 days prior to the experiment. All cells were seeded in their respective growth medium, which is detailed in the section “cell lines”. rCL-11 and rMBL were prepared as serial 2-fold dilutions in supplemented DMEM in pre-plates at concentrations yielding a starting concentration of 20 or 40 µg/ml of protein in the final assay. Subsequently, the prepared protein dilution rows were mixed in 96-well pre-plates with the virus master mix (multiplicity of infection (MOI) of 0.013 for VeroE6 and A549-hACE-2 cells, MOI of 0.1 for Huh7.5 cells, and MOI of 2 for Calu-3 cells, producing an appropriately immunostained cell layer in virus-only control wells as determined in pilot experiments). A SARS-CoV-2 neutralizing mAb HYB 61 (*in-house*^*28*^) was used as a positive neutralization control at a starting concentration of 30 or 40 µg/ml in the final assay. Protein buffers without protein in the respective volumes were included as negative controls. Each experimental condition was tested in four replicate wells for VeroE6, A549-hACE2, and Calu-3 cells, or six replicate wells for Huh7.5 cells. Further, on each plate, at least eight virus-only wells, and at least six blank wells with medium-only were included. The pre-plates were incubated for 2 h at RT. One volume of supplemented DMEM was then added to the pre-plates, and the mixes were added to the 96-well cell plates. The cells were incubated at 37°C and 5% CO_2_ for 48 ± 2 h, except for Calu-3 cells, which were incubated for 72 ± 2 h. After confirmation of cell viability by visual assessment of cellular morphology by light microscopy, cells were fixed and immunostained as described in the section “virus infectious titers”. Finally, the number of SARS-CoV-2 positive cells were determined using the Immunospot series 5 UV analyzer (CTL Europe GmbH) [41], and the percentage of SARS-CoV-2 positive cells in experimental wells relative to the mean number of SARS-CoV-2 positive cells in virus-only wells was calculated.

The rCL-11 supplementation experimental setup in VeroE6 cells was following the same protocol as described above with the following minor changes. After adding the mixes from the pre-plates to the cells, the plates were incubated for 1 h at 37°C and 5% CO_2_. All supernatant was removed, and a new 2-fold dilution of rCL-11 starting at 5 µg/ml was added. Cells were incubated at 37°C and 5% CO_2_ for 48 ± 2 h followed by fixation, immunostaining, and analysis as described above.

### Virus RNA titer quantification

Selected cell culture supernatants from rCL-11 neutralization experiments were prepared for RNA extraction by mixing 1 part sample with 3 parts Trizol LS Reagent (Thermo Fisher Scientific), followed by phase separation with 0.2 volumes of chloroform (Sigma-Aldrich) using 5PRIME Phase Lock Gel Heavy tubes (Quantabio, Beverly, Massachusetts, USA). Subsequently, RNA was purified from the aqueous phase with the Zymo Research RNA clean and concentrator-5 kit (Zymo Research, Irvine, California, USA) according to the manufacturer’s instructions, and RNA was eluted in nuclease-free water (Thermo Fisher Scientific). The quantitative polymerase chain reaction (qPCR) was carried out using TaqMan Fast Virus 1-Step Master Mix (Thermo Fisher) on a LightCycler 96 System (Roche, Basel, Switzerland). Primers and probe were prepared as described elsewhere [40,42] Duplicates of RNA standard (Twist Bioscience, South San Francisco, California, USA) in the range of 10^1^–10^5^ copies/µl, 2.5 µl of sample RNA, and nuclease-free water as a negative control were included for every experiment. Finally, RNA titers were determined by interpolation of cycle threshold values against a standard curve generated using LightCycler 96 software version 1.1.0.1320 (Roche).

## Statistics

Statistical analyses were performed using GraphPad Prism version 9.5 (GraphPad Software Inc, San Diego, California, USA). Normality of the data distribution was tested by Shapiro-Wilks test. Comparison between binding of rCL-11 to full-length WT S protein, RBD, and NTD was performed using ordinary one-way ANOVA following multiple comparisons with Tukey’s correction. Comparisons were performed by comparing the means of the full-length WT, RBD, and NTD to their respective EDTA controls. The C4 deposition was analyzed by ordinary one-way ANOVA test followed by Tukey’s multiple comparison test, where each mean was compared to the mean of every other condition. P-values < 0.05 were considered significant. The differences in rCL-11 binding to SARS-CoV-2 variants were assessed using nonlinear sigmoidal regression 4P curve fitting comparing the HillSlopes. we chose to analyze the Hill slope to compare the mechanistical similarities of the binding as suggested by study in study by Bayarri-Olmos et al [29].

## Results

### CL-11 binds to WT SARS-CoV-2’s S protein with the potential for multiple binding sites

**Fig 1:**
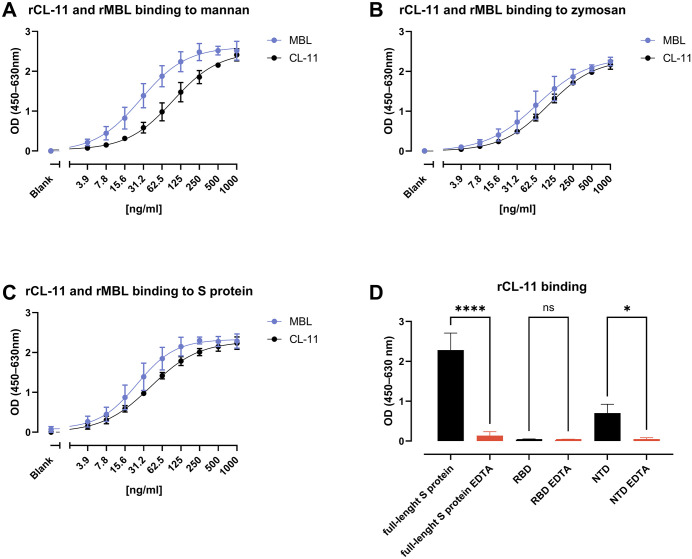
CL-11 interaction with ligands and full-length WT S protein. **(A-C)** Detection of rCL-11 (black) in 2-fold dilutions at the specified concentrations, bound to the ligands (used at 2 µg/ml) mannan (A) and zymosan (B) and full-length WT S protein **(C)**. rMBL (blue) was used as a positive control. Data points represent means of triplicates, and error bars represent SD. **(D)** rCL-11 (0.5 µg/ml, black) binding affinity to different regions of the S protein: full-length WT S protein, RBD, and NTD. S protein was used at 2 µg/ml (11.1 pM), and RBD and NTD were used at the same molar concentration. rCL-11 EDTA (orange) was added as negative control setups. Statistical differences were assessed by ordinary one-way ANOVA with Tukey’s multiple comparison test. ****, p < 0.0001. *, p < 0.05. ns, non-significant. Data are shown as the mean ± SD of three independent replicates.

The S protein of SARS-CoV-2 is heavily glycosylated, providing multiple potential interaction sites for MBL binding [11]. Given the overlap in ligand specificity between MBL and CL-11 [18], the S protein appeared to be a plausible target for CL-11. To validate this hypothesis, binding of rCL-11 to the well-known sugar ligands mannan and zymosan, as well as the full-length WT S protein, was assessed, while rMBL was included as a positive control. rCL-11 demonstrated a slightly lower level of binding to mannan than rMBL (Fig 1A), while the binding to zymosan and the full-length WT S protein appeared comparable between rCL-11 and the rMBL control (**Fig 1B** and **1C**, respectively). The specific regions relevant for CL-11 binding to SARS-CoV-2 were investigated using full-length WT S protein, RBD, and NTD (Fig 1D). rCL-11 efficiently bound to the full-length WT S protein and showed significant binding to the NTD domain. No binding to RBD was detected. This binding pattern suggested a high likelihood of multiple binding sites for CL-11 on the S protein. No binding of rCL-11 was detected in presence of 10 mM EDTA, suggesting that the recognition is calcium-dependent and therefore specifically mediated through the CL-11´s CRD domain. Furthermore, the results are in line with those of Polycarpou et al., who likewise demonstrated that CL‑11 binding to the spike protein was CRD‑mediated, showing >90% inhibition with L‑fucose and a comparable loss of binding in the presence of EDTA [24].

### CL-11 binding remains unaffected across naturally occurring S variants or engineered glycan mutants

**Fig 2:**
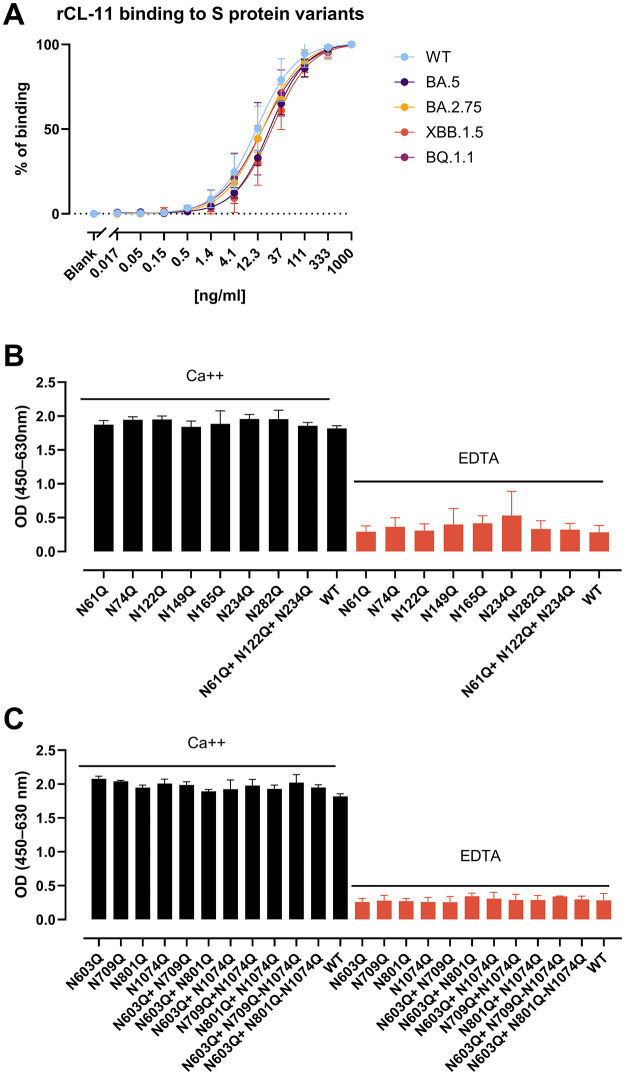
CL-11 binding to full-length S protein from different SARS-CoV-2 variants and S proteins with mutated glycan sites. **(A)** Detection of rCL-11 (0.5 µg/ml) bound to S protein from different SARS-CoV-2 variants (WT, BA.5, BA.2.75, XBB.1.5, and BQ.1.1) coated in 3-fold dilution series at the specified concentrations. WT S protein was used as a positive control and baseline of binding activity. The OD values were normalized to WT binding at each concentration within each of three replicates and expressed as a percentage of WT binding. Data points represent means of triplicates, and error bars represent SD. **(B, C)** rCL-11 binding to the specified S protein N-glycan site mutants from ExpiCHO supernatants (1 µg/ml), immobilized by binding to anti-spike mAb clone 53 (2 µg/ml). rCL-11 (0.5 µg/ml) binding was tested in calcium sufficient (Ca++) and calcium deficient conditions (EDTA). Data are shown as means, and error bars represent ± SD of three independent replicates.

Due to spontaneous mutations and external evolutionary pressure during the COVID-19 pandemic, SARS-CoV-2 constantly accumulated changes that gave rise to different SARS-CoV-2 variants and that possibly modified the glycan shield of their S protein. To investigate whether mutations in SARS-CoV-2 variants result in escape from CL-11 recognition, we evaluated rCL-11 binding affinity using full-length S proteins of the Omicron variants BA.5, BA.2.75, XBB.1.5, and BQ.1.1. By detecting rCL-11 binding to serial dilutions of variant S protein in a direct ELISA setup, we observed high parallelism across all the variants with comparable binding slopes (Fig 2A).

Finally, we aimed to identify which S protein-specific mutations might be crucial for CL-11 binding, thereby predicting the potential for future variants to evade recognition. For this, rCL-11 was tested against a panel of different full-length S proteins, modified at glycan sites proposed to be important for S protein binding by MBL [29]. Notably, none of the mutations seemed to considerably alter the rCL-11 binding compared to the full-length WT S protein (Fig 2B **and** 2C). Experiments with EDTA suggested that most of the binding was calcium-dependent with a low degree of unspecific binding.

## CL-11 binding to S protein activates the lectin and alternative pathways of the complement system

**Fig 3:**
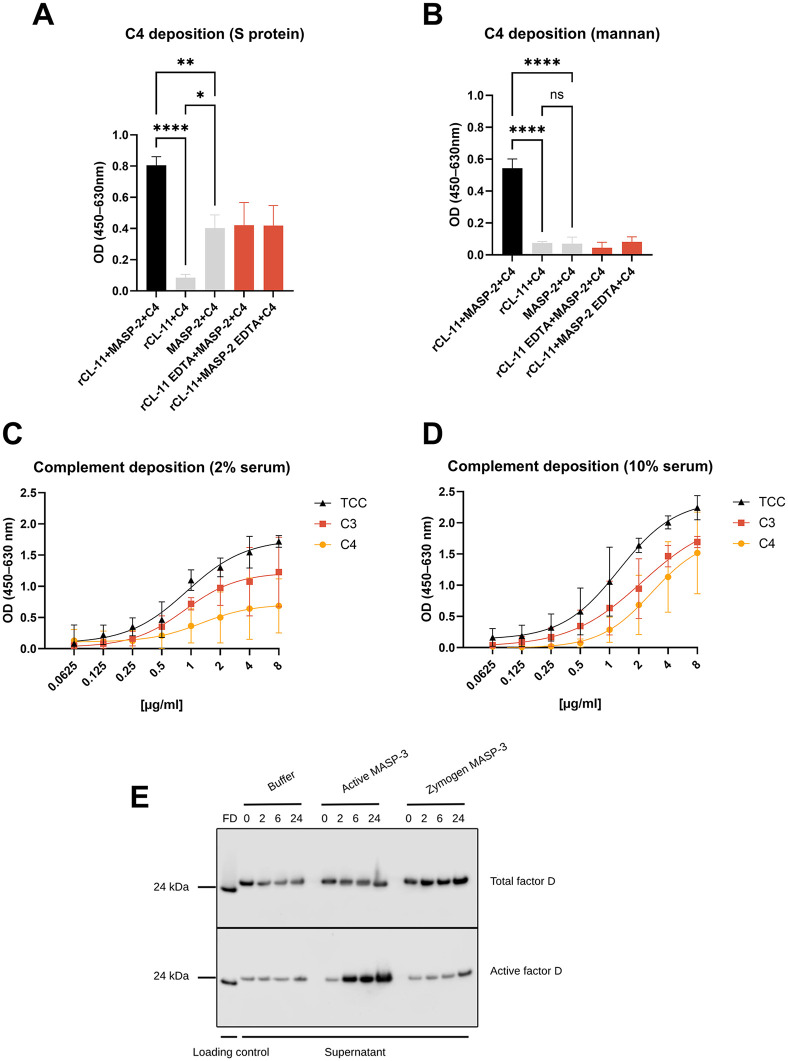
Complement deposition and Factor D activation. **(A, B)** Detection of rC4 deposition on WT S protein (5 µg/ml) (**A**) and mannan used as a positive control (5 µg/ml) (**B**) using rCL-11 (10 µg/ml) and rMASP-2 at 1 µg/ml (rCL-11 + MASP-2 + C4). Negative controls include conditions with buffer instead of rMASP-2 (rCL-11 + C4) or rCL-11 (MASP-2 + C4) and conditions with rCL-11 or rMASP-2 in the presence of EDTA (rCL-11 + MASP-2 EDTA+C4 and rCL-11 EDTA+MASP-2 + C4 respectively). Statistical analysis was done by ordinary one-way ANOVA with Tukey’s multiple comparison test. *, p < 0.05. **, p < 0.01. ****, p < 0.0001 ns, non-significant (also see **Table A** in **S1 Text**). Data are shown as means ± SD of three independent replicates. **(C, D)** CL‑11–dependent complement activation using MBL‑deficient serum at 2% (C) and 10% (D) on WT S‑protein–coated plates (2 µg/ml). CL‑11 was applied in a 2‑fold dilution series starting at 8 µg/ml. Deposition of C4b, C3b, and TCC was detected using specific monoclonal antibodies. Values from the control wells (no CL-11) were subtracted from the experimental values to account for CL‑11‑independent complement activity. Data are shown as means ± SD of three independent replicates. **(E)** Detection of Total and Active Factor D by WB. Pro-Factor D was incubated with WT S protein (1 µg/ml) pre-bound by rCL-11 (1 µg/ml), in the presence of buffer, active MASP-3 (1 µg/ml), or MASP-3 zymogen (1 µg/ml). Samples were collected at 0, 2, 6, and 24 hours. Western blot analysis was performed using monoclonal antibodies that detect either total Factor D (Pro-Factor D + Active Factor D) or the active form specifically. MASP-3 zymogen and buffer-only conditions served as negative controls. The figure shows a representative blot from three independent replicates. Quantifications of band intensities are shown in **Fig B** in **S1 Text**. FD, Factor D control.

Complement activation is an important effector function of CL-11 in the defense against pathogens [20]. Therefore, the ability of CL-11 to deposit C4 protein—the first step in the activation of the complement cascade via the lectin pathway—on the surface of WT S protein was investigated using rMASP-2 **(Fig 3A)**. rCL-11 could mediate deposition of C4b on the S protein via rMASP-2. However, approximately 50% of the signal was retained in the presence of EDTA, and when rCL-11 was removed from the assay completely. The signal disappeared when rMASP-2 was removed from the assay **(Fig 3A)**. In contrast, in the mannan control setups, no signals were detected in any of the negative controls **(Fig 3B)**. The full statistical results can be found in **Fig B** in **S1 Text**. These results suggest in addition to a rCL-11 and rMASP-2 dependent C4 deposition a direct interaction between rMASP-2 and S protein that is not calcium-dependent but retains the ability to cleave C4. Building on these findings, we next assessed the ability of CL‑11 to drive deposition of C4b, C3b, and formation of the terminal complement complex (TCC) via the lectin pathway (**Fig 3C and 3D**). rCL‑11 bound to WT S protein and, upon incubation with MBL‑deficient serum at either 2% (**Fig 3C**) or 10% (**Fig 3D**) induced deposition of C4, C3, and TCC in a concentration‑dependent manner. These deposited components represent the hallmarks of full complement activation.

Besides the interaction of CL-11 with MASP-2, it was also shown that CL-11 interacts with MASP-3 [43]. Through interaction with MASP-3, CL-11 can connect the lectin and alternative pathways by activation of Factor D [22]. **Fig 3E** illustrates the ability of rCL-11 bound to the full-length WT S protein to bind active rMASP-3 and cleave Pro-Factor D into active Factor D using an ELISA/WB setup. Samples were collected at 0, 2, 6, and 24 h of incubation, showing an increasing intensity of the active Factor D bands over time when presented with active MASP-3. Based on the quantification of the band intensity (**Fig B** in **S1 Text**), the percentage of Active Factor D in presence of active MASP-3 was ranging from approximately 5% at 0 h to 40% at 24 h. Pro-Factor D without rMASP-3 and rMAPS-3 zymogen were used as negative controls. Only the active rMASP-3, after interaction with rCL-11, could activate Factor D.

**CL-11 interferes with S protein-receptor binding and neutralizes SARS-CoV-2 in an antibody-independent manner protecting SARS-CoV-2 permissive cells from infection**

**Fig 4:**
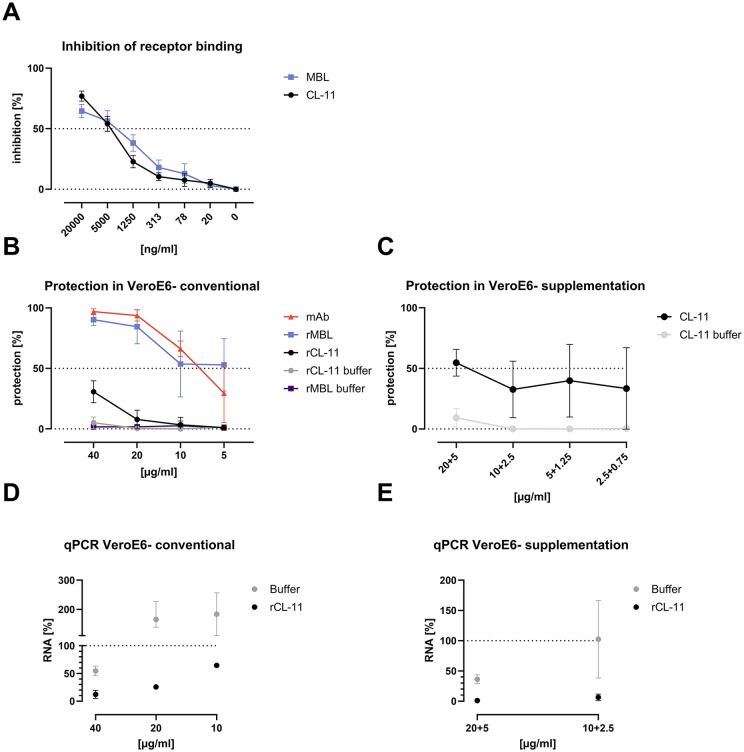
Receptor-binding inhibition and SARS-CoV-2 neutralization in VeroE6 cell-based assays. **(A)** In an ELISA-based S protein-receptor binding assay, WT S protein (1 µg/ml) binding to ACE-2 receptor (1 µg/ml) was detected after fluid phase interaction with rCL-11 or rMBL in 2-fold dilution series at the specified concentrations. The percentage of inhibition was calculated by comparing the receptor binding signal in the presence of rCL-11 or rMBL to the signal from negative control wells. Data are shown as means ± SD of three independent replicates. **(B)** Neutralization of infectious SARS-CoV-2 following incubation with two-fold dilutions of rMBL (blue), rCL-11 (black), or mAb clone 61 (orange) starting at the 40 µg/ml using VeroE6 cells in a conventional experimental setup. **(C)** Neutralization of infectious SARS-CoV-2 following incubation with rCL-11 (two-fold, starting at 20 µg/ml) and a subsequent rCL-11 supplementation (two-fold, starting a 5 µg/ ml) 1 h post-infection (black) in VeroE6 cells. (B-C) rCL-11 buffer (gray) in corresponding dilution was used to monitor buffer-mediated effects on the cells. The percentage of protected cells was determined from the number of SARS-CoV-2 infected cells in experimental wells compared to the number of infected cells in virus-only control wells after anti-S protein immunostaining. Data are shown as means ± SD of four replicates. **(D)** Percentage change in SARS-CoV-2 RNA copies/µl in selected rCL-11 wells from the experiment in (B) relative to the virus-only wells. **(E)** Percentage change in SARS-CoV-2 RNA copies/µl in selected wells from the experiment in (C) relative to the virus-only wells. **(D, E)** The mean number of RNA copies/µl measured in virus-only wells was set to 100% (dotted line). The lower limit of detection of the assay was 4 RNA copies/µl. Results are shown as means ± SD of two replicates.

Another major effector function of collectins against pathogens is their antibody-independent neutralization potential [44]. Therefore, the capability of rCL-11 to inhibit S protein binding to the ACE-2 receptor and to inhibit SARS-CoV-2 infection of permissive cells were assessed in receptor binding and neutralization assays, respectively. First, the influence of rCL-11 on binding of WT S protein to the ACE-2 receptor was investigated in an ELISA-based receptor-binding assay (Fig 4A**).** Preincubation of S protein with serial dilutions of rCL-11 or the control rMBL resulted in comparable concentration-dependent inhibition of S protein-ACE-2 binding.

To assess the implications of the observed inhibition of S protein-ACE-2 binding in a more physiological setup, we next carried out cell-based SARS-CoV-2 neutralization assays. In these experiments, the influence of rCL-11 on the capacity of the original SARS-CoV-2 to infect four different cell lines was investigated.

Using a direct neutralization assay, we first investigated African green monkey kidney-derived VeroE6 cells, which are widely used for SARS-CoV-2 infection experiments. In these cells, preincubation of SARS-CoV-2 with rCL-11 protected 40% of cells at 40 µg/ml, however, at lower concentrations its activity rapidly declined, evaluated based on immunostaining for S protein (**Fig 4B**). In comparison, rMBL was slightly more effective, protecting more than 50% of cells at all tested dilution points. An S protein specific neutralizing mAb, used as a positive control, efficiently protected the cells from infection.

Because there is a constant production of CL-11 by the lung epithelium *in vivo*, we employed an additional experimental setup in VeroE6 cells. Here, following incubation of virus/rCL-11 mixes on the cells, the inoculum was removed, and fresh medium with extra rCL-11 was supplemented (Fig 4C). In this rCL-11 supplementation setup, rCL-11 showed increased capacity to protect VeroE6 cells from infection. At the highest concentration (20 + 5; 20 µg/ml initial dose, 5 µg/ml supplementation), more than 50% of cells were protected from viral infection, while at 20 µg/ml only 10% of cells were protected in the conventional experimental setup; further, close to 50% protection was retained at lower rCL-11 dilutions.

To enhance the sensitivity and dynamic range of SARS-CoV-2 detection, the total viral RNA was analyzed in selected samples at the highest concentrations of rCL-11 in the conventional (Fig 4B) and the supplementation (Fig 4C) neutralization experiments and compared to viral RNA in the positive control virus-only wells. In the conventional setup, RNA copy numbers were significantly reduced in wells with rCL-11 compared to in virus-only wells (Fig 4D), and this change was concentration dependent. The protective effect of rCL-11 was further enhanced in the supplementation experiment (Fig 4E**),** with >80% reduction comparing RNA copy numbers at the highest concentration (20 + 5 µg/ml) of rCL-11 to that in virus-only wells.

**Fig 5:**
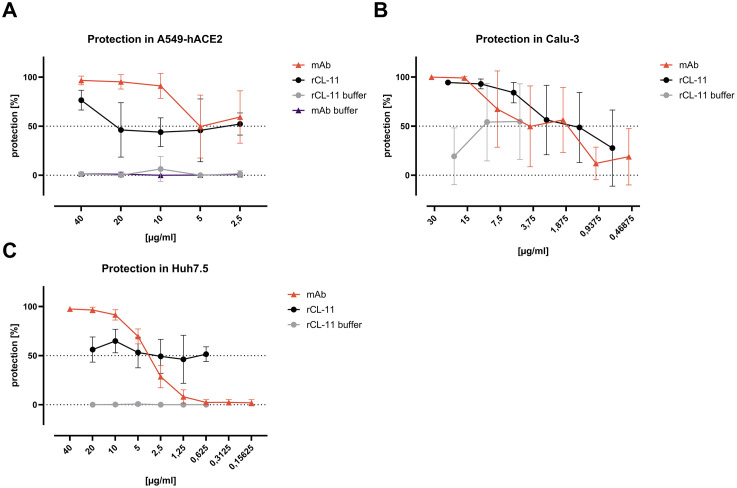
SARS-CoV-2 neutralization assays in A549-hACE-2, Calu-3, and Huh7.5 cells. **(A-C)** Neutralization of infectious SARS-CoV-2 following incubation with two-fold dilutions of rCL-11 (black) in A549-hACE-2 cells (starting at 40 µg/ml) **(A)**, Calu-3 cells (starting at 20 µg/ml) **(B)**, and Huh7.5 cells (starting at 20 µg/ml) (C) using the conventional experimental setup. The mAb clone 61 was used as a neutralization control (orange) and rCL-11 buffer (gray) in corresponding dilutions was used to monitor buffer-mediated effects on the cells. The percentage of protected cells was determined from the number of SARS-CoV-2 infected cells in experimental wells compared to the number of infected cells in virus-only control wells after anti-S protein immunostaining. Data are shown as means ± SD of four (A549-hACE-2 and Calu-3 cells) or six (Huh7.5 cells) replicates.

We then investigated three human cell lines for the capacity of rCL-11 to protect against SARS-CoV-2 infection using the direct neutralization assay. In human lung-derived A549 cells engineered to express human ACE-2 (A549-hACE-2 cells), rCL-11 achieved protection levels exceeding 60% at the highest concentration but plateaued around 50% at lower concentrations (Fig 5A). Importantly, in human lung-derived Calu-3 cells, which naturally express ACE-2, rCL-11 conferred more than 80% protection at the highest concentrations, with the effect titrating down at lower concentrations (Fig 5B). In human liver-derived Huh7.5 cells, also naturally expressing ACE-2, rCL-11 provided around 50% protection at all tested concentrations (Fig 5C).

In addition, in an indirect neutralization assay, we showed that neutralization of SARS-CoV-2 by CL-11 followed by infection of Calu-3 cells resulted in a decreased production of infectious virus by these cells (**Text A** and **Fig C** in **S1 Text**).

## Discussion

In the current study, we analyzed CL-11’s interaction with SARS-CoV-2 and explored its contributions to viral defense. We showed that CL-11 binds to the full-length S protein of the SARS-CoV-2, following a similar pattern as MBL. Moreover, the robust binding, coupled with the possibility of multiple binding sites, may make it difficult for future viral variants to fully escape CL-11 recognition, potentially reducing the effectiveness of escape mutations. After binding, CL-11 interacted with MASP-2 and MASP-3, activating both the lectin and alternative pathways of the complement system. In addition to complement activation, CL-11 inhibited S protein-receptor binding and SARS-CoV-2 in an antibody independent manner, protecting permissive cells from infection.

Despite structural similarities and overlap in ligand recognition, CL-11’s CRD shares only 25–32% sequence identity with that of other collectins such as MBL [18]. Notably, CL‑11 possesses an extended binding site within its CRD, which allows for broader ligand diversity but may result in lower binding affinity. When compared to MBL, CL-11 exhibited similar levels of lectin activity towards mannan, zymosan, and S protein, with MBL taking a minor lead. In VeroE6 cells, however, MBL demonstrated markedly higher SARS-CoV-2 neutralization activity than CL‑11, protecting more than twice the number of cells at both 40 and 20 µg/ml. The high concentrations of MBL neutralized nearly as effectively as the monoclonal antibody used as a positive control.

The differing performance between CL‑11 and MBL may reflect distinct binding site preferences, with CL‑11 potentially targeting regions less critical for viral entry. However, the most likely explanation is, that CL‑11 may bind with lower avidity due to differences in its oligomerization. This is supported by the greater prevalence of higher-order oligomeric forms in the purified recombinant MBL preparation (**Fig A.B** in **S1 Text**) compared to the recombinant CL‑11 used in our study. CL-11 is notoriously difficult to produce recombinantly in stable higher-order oligomeric forms [ 18]. Therefore, we use recombinant CL-11 directly from clarified ExpiCHO supernatant, which contains a mixture of lower and higher oligomeric variants as confirmed by WB (**Fig A.A** in **S1 Text**). In addition, the inherent heterogeneity of both MBL and CL-11 preparations precludes accurate molar quantification (**Fig A** in **S1 Text**). Consequently, comparisons between MBL and CL-11 in this study are based on protein concentrations rather than molar concentrations. MBL was mainly included as a positive control of choice, and the experiments should not be interpreted as direct comparisons of binding and neutralization potential between MBL and CL-11. Future structural comparisons between CL-11 and MBL would be of interest to the field.

The variance in CL-11 production could also explain the difference between our results and the results reported by Stravalaci et al., who found only negligible binding of CL-11 to SARS-CoV-2 S protein. This discrepancy could be attributed to their protein being expressed as a purified single trimer. Moreover, their protein was produced in a wheat germ expression system likely altering post-translational modifications important for correct folding and oligomerization of the protein [11].

Taking a closer look at the binding distribution of CL-11 in our study, we found that the protein bound primarily to the full-length S protein with diminished but significant binding to the NTD region. No binding was detected to the RBD alone. This binding pattern is in line with the previous research of MBL binding [29] as well as studies of the S protein structure reporting only a limited number of glycans on the surface of RBD [45]. Moreover, there were no significant differences in CL-11 binding to naturally occurring S protein variants, i.e., WT S protein and the omicron variants BA.5, BA.2.75, XBB.1.5, and BQ.1.1., which were prevalent during 2023 (https://covariants.org/). This result suggests that naturally occurring SARS-CoV-2 variants fail to evade CL-11 binding despite harboring a number of mutations across different S protein regions [46]. This is in line with the conclusion of Polycarpou et al., who observed no difference in binding affinity of CL-11 to Wuhan like S protein and Delta S protein, respectively [24].

To identify the specific glycan sites relevant for CL-11 binding, we used a selection of artificially mutated recombinant S proteins. We tested 11 point-mutations in S protein glycans that were *in silico* predicted to determine collectin’s binding [11] and that were experimentally verified [45]. However, the binding of CL-11 remained unchanged by these mutations tested singly and in combination. This suggested that no single glycan sites were responsible for CL-11’s binding in contrast to a previous model of collectin binding to SARS-CoV, which identified a single glycan site (N330) to be the determinant for MBL’s binding [7]. On the contrary, CL-11 binding to S protein appeared to be robust involving multiple binding sites, implying that future variants would need to undergo significant changes to escape from CL-11 recognition. This is particularly relevant since some glycan site mutations appear to have deleterious effects on S protein stability [29].

CL-11 bound to S protein was able to activate both the lectin and alternative complement pathways via association with MASP-2 and MASP-3, respectively. This agrees with earlier studies showing lectin pathway activation when CL‑11, MBL, or normal human serum were exposed to SARS‑CoV‑2 [24,47]. Importantly, our work extends these findings by demonstrating that CL‑11 can also directly activate the alternative pathway, highlighting its potential role as a key link between lectin and alternative pathway activation.

Generally, complement activation promotes pathogen opsonization, release of anaphylatoxins, and recruitment of immune cells such as neutrophils, macrophages, and mast cells to the site of infection. These processes contribute to antiviral defense. On the other hand, an increasing body of evidence suggests, that complement activation driven by SARS-CoV-2 can contribute to severe COVID-19 and risk of hospitalization [48]. Ma et al. reported that levels of complement activation markers, particularly C5a, are significantly higher in patients hospitalized with COVID-19 compared to those hospitalized with influenza [49]. Elevated C5a activity can cause excessive activation of neutrophils and monocytes, which might result in immunopathology [50]. Markers of the alternative pathway, including Factor B and Factor D, have also been implicated in endothelial injury and enhanced coagulation in severe cases of SARS-CoV-2 infection [49,51,52].

CL‑11’s dual ability to activate both the lectin and alternative complement pathways suggests a protective role against SARS‑CoV‑2 infection, but at the same time raises the possibility of contributing to epithelial injury and inflammatory sequelae. While our in vitro system shows that CL‑11 can trigger complement activation mediated by the S protein in a concentration-dependent manner, it does not allow us to determine its true physiological significance. To clarify the potential immunopathology of CL‑11–driven complement activity, investigations in relevant *in vivo* models and clinical studies will be required. Such analyses lie beyond the scope and practical possibilities of the present study.

In addition to activating the complement system, CL-11 inhibited S protein-ACE-2 receptor binding, most likely by interacting with the surface of the S protein and thereby preventing its physical engagement with ACE-2. This inhibition occurred in a concentration-dependent manner, with a slope comparable to that observed for the MBL control. Furthermore, in live-virus neutralization assays, CL-11 protected four different SARS-CoV-2–permissive mammalian cell lines from infection. These cell lines originate from distinct tissues and differ markedly in their natural expression of the critical viral receptor ACE-2. Specifically, A549-hACE-2 and Calu-3 cells are derived from human lung tissue and VeroE6, Huh7.5, and Calu-3 cells naturally express ACE-2. Such inherent differences, together with the varying MOIs required for efficient infection of each cell line, likely account for the observed variation in CL-11 neutralization efficacy. Importantly, across all models tested, CL-11 consistently conferred protection against SARS-CoV-2 infection, underscoring its potential role as a protective lectin at the cellular level *in vitro*.

These results are in contrast with the study by Polycarpou et al. [24] who did not observe any CL-11 mediated neutralization in a similar setup but in fact observed increased SARS-CoV-2 production in the presence of 10 µg/ml rCL-11. This discrepancy between our results and those by Polycarpou et al. [24] is most likely caused by differences in protein composition. Both studies are using ExpiCHO produced recombinant CL-11. However, Polycarpou et al. [24] further purify the protein, yielding CL-11 complexes of 105 KDa representing a single trimer. In contrast, our protein is in clarified cell culture supernatant and exhibits a diversity of oligomeric forms including single trimers at around 100 KDa, as well as dimers, trimers, and tetramers of trimers (**Fig A** in **S1 Text**). This is consistent with findings by Hansen et al., who observed robust CL‑11‑mediated neutralization of other viruses at 25 µg/ml and proposed that native CL‑11—which frequently oligomerizes into higher‑order structures, including hexamers of trimers—may be more effective than lower-order oligomeric forms [18]. Their conclusions align with previous studies showing that collectin oligomerization is important for antiviral function [53]. Nevertheless, we cannot entirely exclude that the discrepancy between our results and those by Polycarpou et al. [24] is caused by differences in the applied assays. Importantly, across all our experiments, we consistently observed that CL-11-mediated neutralization of SARS‑CoV‑2.

Despite CL-11 being potentially a less potent viral neutralizer compared to MBL, one of its major advantages might stem from differences in tissue expression between the two molecules. According to available databases [23], human MBL is exclusively expressed in the liver and is released directly into circulation. Therefore, it could be effective against potential viral spread in the blood stream. In contrast, CL-11 is expressed in many tissues, including the lungs [23,24]. The lung epithelium surfaces are the main entry points for SARS-CoV-2 into the body [54,55]. Therefore, proteins expressed directly on the epithelium surface might be of greater importance for initial viral defense. CL-11 on-site production in the lungs can likely be increased by signaling through proinflammatory cytokines [24,25]. Thus, the constant production of CL-11 may increase its activity. Indeed, when we supplemented the initial dose of CL-11 with additional protein the effectiveness of neutralization increased.

Additionally, it is important to consider potential interactions of CL‑11 with other collectins such as MBL and CL‑10. CL‑10 can form heterocomplexes with CL‑11, and these complexes have been detected in circulation, although they are not typically present at epithelial surfaces where CL‑11 predominates [23]. In contrast, MBL has potentially higher binding avidity but is generally absent from the lung epithelium under steady‑state conditions [23]. Thus, locally produced CL‑11 oligomers are likely to act as the primary line of defense in the respiratory tract. Nevertheless, during inflammation, increased vascular permeability may allow MBL and CL‑10/11 complexes to enter the tissue, raising the possibility of competition, altered binding properties, or synergistic activity. While our study specifically addresses the role of CL‑11 alone, future work should explore how these interactions shape complement activation and antiviral protection *in vivo*.

## Conclusion

This study establishes CL-11 as a key innate immune effector in the defense against SARS-CoV-2 infection. We demonstrate that CL-11 binds to the viral S protein, activates the complement system, inhibits S protein-receptor binding, and neutralizes the virus in an antibody-independent manner. Its ability to recognize multiple SARS-CoV-2 variants suggests a robust, broad-spectrum, antiviral function. These findings expand our understanding of complement-driven antiviral defense and highlight CL-11 as a potential target for novel antiviral strategies. Further investigation into its mechanisms and therapeutic applications could pave the way for innovative approaches to combat respiratory viral infections.

## Supporting information

S1 Data Raw data used to generate Fig 1. (XLSX)

S2 Data Raw data used to generate Fig 2. (XLSX)

S3 Data Raw data used to generate Fig 3. (XLSX)

S4 Data Raw data used to generate Fig 4. (XLSX)

S5 Data Raw data used to generate Fig 5. (XLSX)

S6 Data Raw data used to generate Fig C in S1 Text. (XLSX)

S1 Text **Additional supporting experiments, methods, and extended statistical analysis.** This document contains: (**Text A**) supplementary methods related to Fig C; (**Table A**) complete statistical analysis of deposition of C4 to S protein and mannan shown in Fig 3A and 3B; (**Figure A**) size distribution of the recombinant proteins used in the study.; (**Fig B**) Quantification of Pro-Factor D activation; and (**Fig C**) indirect SARS-CoV-2 neutralization assay.
(DOCX)
