# Supplementary material for: Collectin-11, a complement pattern recognition molecule, mediates pulmonary SARS-CoV-2 neutralization and protection
Source: PLoS Pathog. 2026 May 13;22(5):e1014216. doi: 10.1371/journal.ppat.1014216 (PMC13197076; doi:10.1371/journal.ppat.1014216)

**S1 Text**

**Text A: Indirect virus neutralization assay.**

Calu‑3 cells were seeded at 200,000 cells per well in a 48-well plate two days prior to the experiment. Recombinant CL‑11 (rCL‑11) (clarified supernatant) was prepared as a 2‑fold dilution series, starting at a concentration of 20 µg/ml. Each rCL‑11 dilution was mixed with virus master mix (corresponding to an MOI of 0.5 or 1). The mixtures were incubated for 1 h at 37 °C and 5% CO₂. After incubation, the rCL‑11/virus mixtures were added to Calu‑3 cells, including virus‑only (VO) (no pre-incubation with CL-11) and blank controls (medium only), and incubated for 1 h at 37 °C and 5% CO₂. The inoculum was then removed, and the cells were washed once with pre‑warmed PBS, and pre‑warmed DMEM was added. After 24 h at 37 °C and 5% CO₂, supernatants from the Calu‑3 cultures were collected. The 50% Tissue Culture Infectious Dose (TCID_50_) values of the supernatants were determined as described in the section *Virus infectious titers* in Materials and Methods.

**Table A: Complete statistical analysis of deposition of C4 to S protein and mannan shown in Figure 3A and 3B**. Statistical analysis was done by ordinary one-way ANOVA and the Tukey’s multiple comparison test.

| **Tukey's multiple comparison test** | **Summary** | **Adjusted P value** |
| --- | --- | --- |
| **S protein (Fig. 3A)** |  |  |
| rCL-11+ MASP-2+ C4 **vs.** rCL-11+ C4 | **** | <0.0001 |
| rCL-11+ MASP-2+ C4 **vs.** MASP-2+ C4 | ** | 0.0037 |
| rCL-11+ MASP-2+ C4 **vs.** rCL-11 EDTA+ MASP-2+ C4 | ** | 0.0052 |
| rCL-11+ MASP-2+ C4 **vs**. rCL-11+ MASP-2 EDTA+ C4 | ** | 0.0049 |
| rCL-11+ C4 **vs**. MASP-2+ C4 | * | 0.018 |
| rCL-11+ C4 **vs.** rCL-11 EDTA+ MASP-2+ C4 | * | 0.0125 |
| rCL-11+ C4 **vs.** rCL-11+ MASP-2 EDTA+ C4 | * | 0.0132 |
| MASP-2+ C4 **vs.** rCL-11 EDTA+ MASP-2+ C4 | ns | 0.9991 |
| MASP-2+ C4 **vs.** rCL-11+ MASP-2 EDTA+ C4 | ns | 0.9995 |
| rCL-11 EDTA+ MASP-2+ C4 **vs.** rCL-11+ MASP-2 EDTA+ C4 | ns | >0.9999 |
| **Mannan (Fig. 3B)** |  |  |
| rCL-11+ MASP-2+ C4 **vs.** rCL-11+ C4 | **** | <0.0001 |
| rCL-11+ MASP-2+ C4 **vs.** MASP-2+ C4 | **** | <0.0001 |
| rCL-11+ MASP-2+ C4 **vs.** rCL-11 EDTA+ MASP-2+ C4 | **** | <0.0001 |
| rCL-11+ MASP-2+ C4 **vs**. rCL-11+ MASP-2 EDTA+ C4 | **** | <0.0001 |
| rCL-11+ C4 **vs**. MASP-2+ C4 | ns | 0.9998 |
| rCL-11+ C4 **vs.** rCL-11 EDTA+ MASP-2+ C4 | ns | 0.8679 |
| rCL-11+ C4 **vs.** rCL-11+ MASP-2 EDTA+ C4 | ns | 0.9993 |
| MASP-2+ C4 **vs.** rCL-11 EDTA+ MASP-2+ C4 | ns | 0.9255 |
| MASP-2+ C4 **vs.** rCL-11+ MASP-2 EDTA+ C4 | ns | 0.9947 |
| rCL-11 EDTA+ MASP-2+ C4 **vs.** rCL-11+ MASP-2 EDTA+ C4 | ns | 0.762 |

*, p < 0.05., **, p < 0.01. ****, p < 0.0001. ns, non-significant.

**Figure A: Size distribution of the recombinant proteins used in the study**. **(A)** Western blot showing the multimerization states of the clarified CL-11 supernatant used for the experiments throughout the paper. The CL-11 is detected using *in-house* monoclonal anti-CL-11 antibody HYB 16. The different oligomeric forms of CL-11 are distributed according to their molecular weights; in KDa starting from monomer of trimers at around 90 KDa with other prominent structures including dimers of trimers (130 KDa), trimers of trimers (160 KDa), and possibly higher structures that are however beyond the detection scope of this gel. **(B)** Electrophoresis and Coomassie blue staining of selected purified recombinant proteins, from the left: S protein, RBD, NTD, and MBL in non-reducing (left) and reducing conditions (right, 2x DSS). Protein sizes in reducing conditions were 150 KDa for S protein trimer, 37 KDa for RBD, and 50 KDa for NTD. In non-reducing conditions, the size of MBL was close to the upper limit of detection, with a range of tetramers to hexamers of trimers expected to be the most prevalent.

**
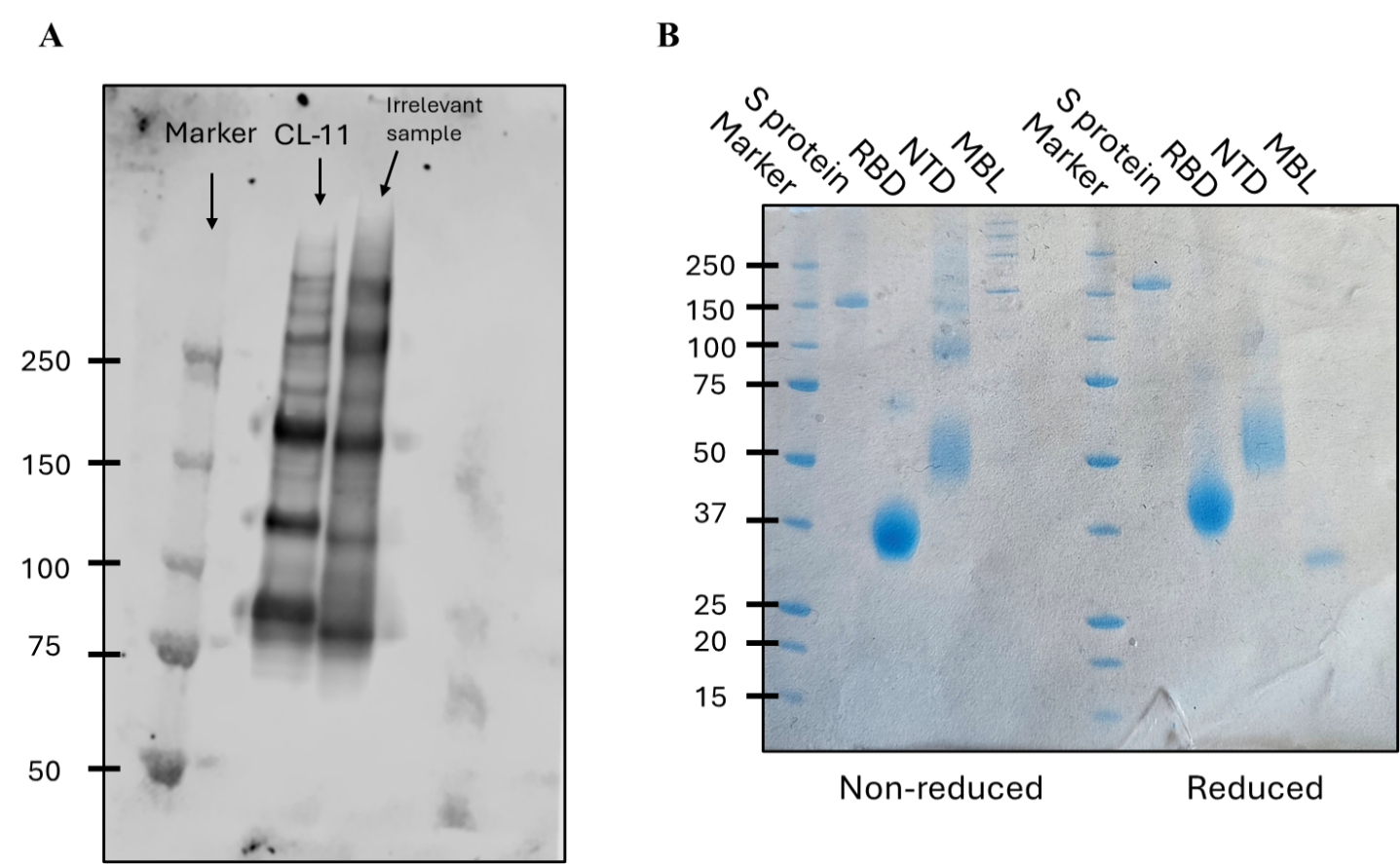
**

**Figure B: Quantification of Pro-Factor D activation. (A)** The proportion of Active Factor D band signal intensity relative to Total Factor D band signal intensity was calculated based on the intensity values from the WB in Figure 3E and showed as percentage of activation for each respective condition in Figure 3E. FD, Factor D. Act. MASP-3, Active MASP-3. Zym. MASP-3, Zymogen MASP-3.


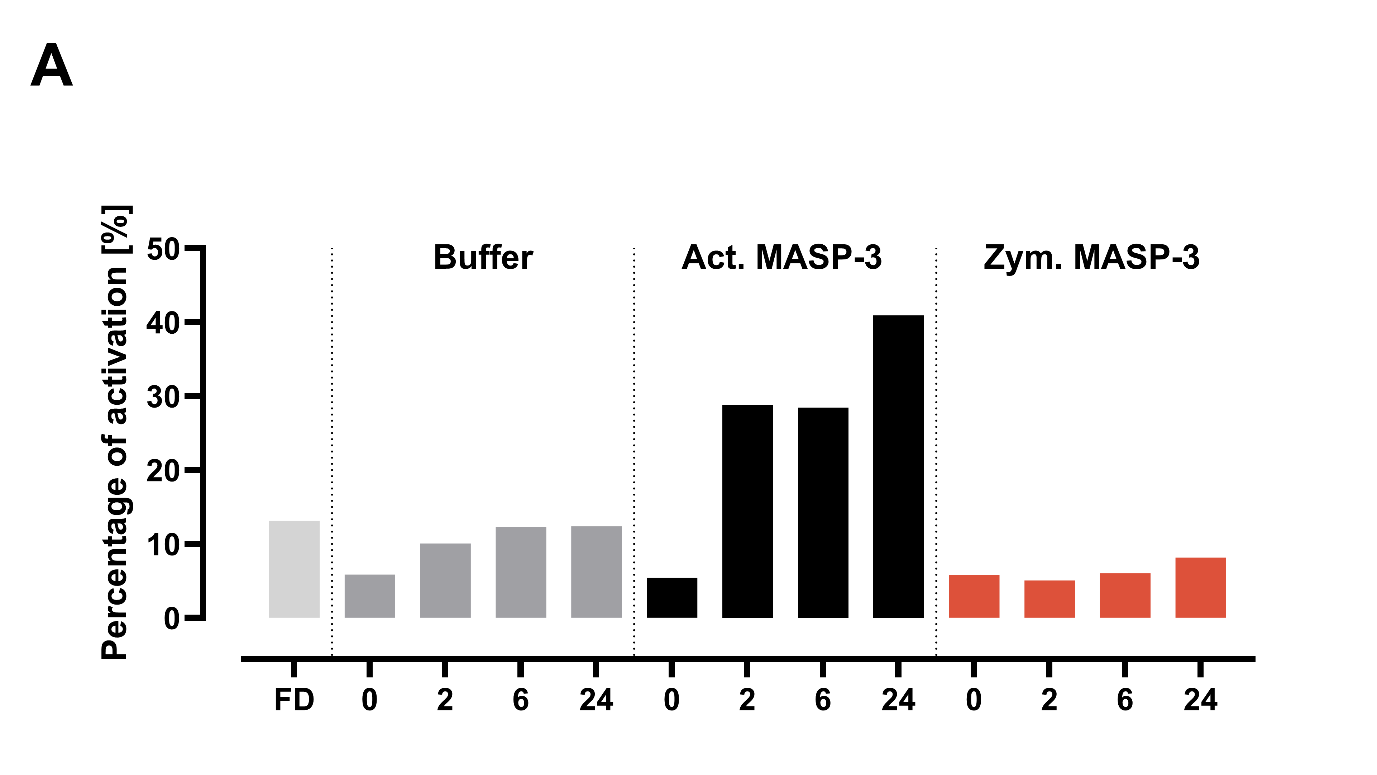


**Figure C:** **Indirect SARS-CoV-2 neutralization assay**. (**A, B**) Neutralization of infectious SARS-CoV-2 following incubation with two-fold dilutions of rCL-11 (starting at 20 µg/ml) in Calu-3 cells using MOIs of 0.5 (**A**) and 1 (**B**). In the indirect assay, virus-protein mixtures were replaced with fresh medium after 1 h. After 24 h, supernatants from the Calu-3 cells were collected, and infectious virus titers were determined in TCID₅₀ assays using VeroE6 cells. Data are presented as infectivity titers (log₁₀ TCID₅₀/ml) of supernatants collected from Calu-3 cells inoculated with CL-11 pre-incubated virus (orange bars) or virus not pre-incubated with CL-11 (black bars), each condition was tested in singular. TCID₅₀, 50% tissue culture infectious dose. MOI, multiplicity of infection. VO, virus only.


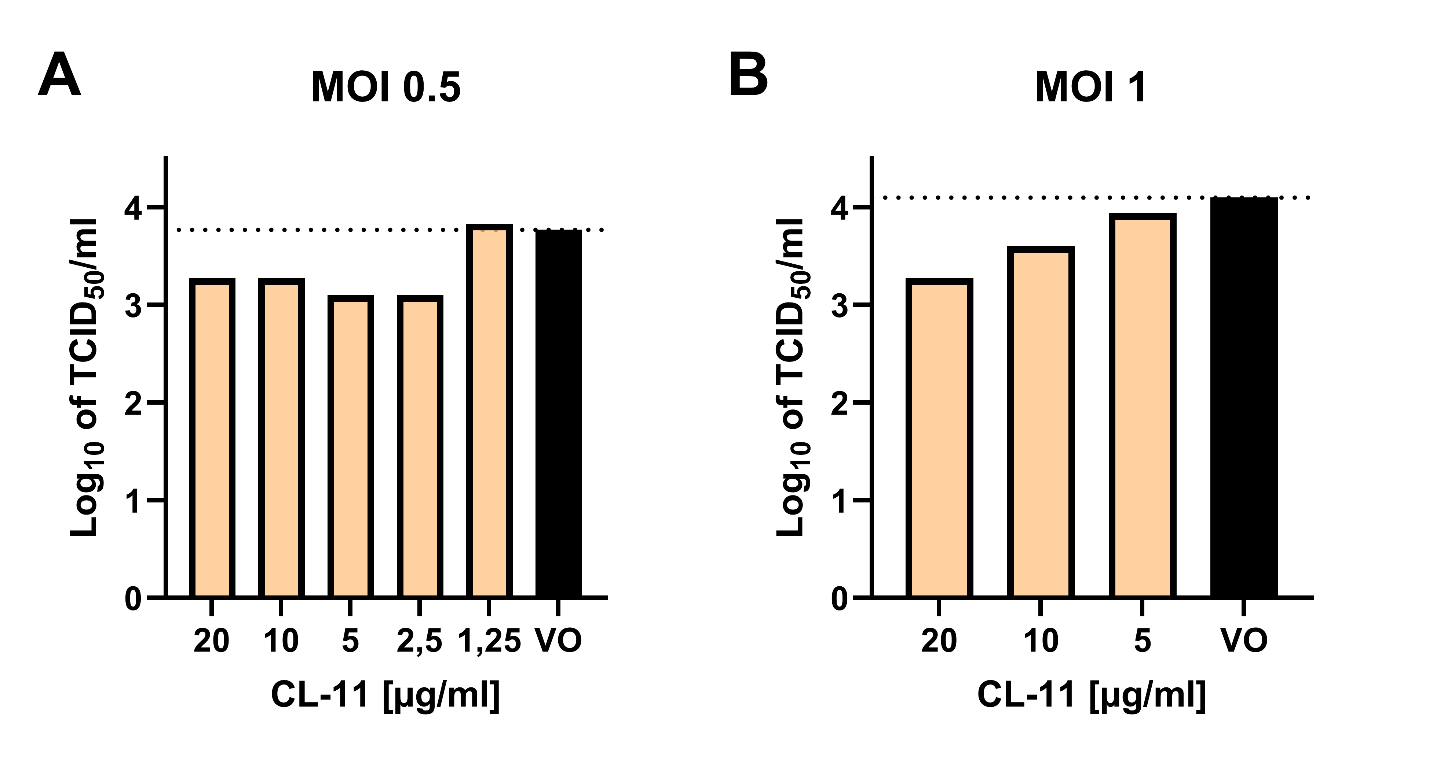

Supplement: S1 Text — Additional supporting experiments, methods, and extended statistical analysis. This document contains: (Text A) supplementary methods related to Fig C; (Table A) complete statistical analysis of deposition of C4 to S protein and mannan shown in Fig 3A and 3B; (Figure A) size distribution of the recombinant proteins used in the study.; (Fig B) Quantification of Pro-Factor D activation; and (Fig C) indirect SARS-CoV-2 neutralization assay. (DOCX) [file ppat.1014216.s007.docx]
